# Beyond COVID-19 Pandemic: An Integrative Review of Global Health Crisis Influencing the Evolution and Practice of Corporate Social Responsibility

**DOI:** 10.3390/healthcare9040453

**Published:** 2021-04-12

**Authors:** Henry Asante Antwi, Lulin Zhou, Xinglong Xu, Tehzeeb Mustafa

**Affiliations:** 1 Centre for Health and Public Policy Research, Jiangsu University, 301 Xuefu Road, Zhenjiang 212013, China; lulinzhou@yahoo.com (L.Z.); 5103140204@stmail.ujs.edu.cn (T.M.); 2 School of Management, Jiangsu University, 301 Xuefu Road, Zhenjiang 212013, China; 1000004932@ujs.edu.cn

**Keywords:** CSR, implication, public, health, evolution, COVID-19

## Abstract

**Background:** Global health crisis continues to drive the dynamics of corporate social responsibility (CSR) across industries with self-perpetuating momentum. From a historical point of view, more than a century of immense corporate fecundity has formed the ecological conditions and shaped current understanding of the effect of public health on CSR. This study sought to examine the extent to which companies are able to balance their business interest with social interest through health-related CSR and how knowledge of them can help explain the potential impact of COVID-19. **Method:** This study employs a narrative review of current literature; however, the integrative strategy was combined with the Preferred Reporting Items for Systematic reviews and Meta-Analyses (PRISMA) checklist to rigorously select the necessary articles for proper integrative synthesis. **Results:** We note that in the pursuit of their social responsibility, corporate enterprises struggle to balance the interest of society and their own interest. Genuine CSR activities such as donations are often undermined by unbridled and excessive desire to draw society on themselves to reap economic benefits are largely dominated by the need to advance. There are signals that enterprises might see COVID-19-related CSR as an entry door to increase corporate influence thereby commercializing the pandemic. **Conclusions:** The impact of COVID-19 on CSR is epochal. There is a moral obligation for enterprises to reform current risk assessments and collaborate more deeply with state agencies to invest in the health and safety inspections at the world place. CSR strategies must be proactive to endure other unknown pandemics with equal capacity to disrupt business operations. Companies must create innovative and regular activities to educate its stakeholders to become more committed to safeguarding future enterprise-based defense mechanism needed to diagnose, protect, treat, and rehabilitate victims and those threatened by pandemics and other emergencies that affect the stability of an organization to reduce its cost and protect revenue.

## 1. Introduction

Nowadays, an ever-increasing number of enterprises recognize the need to voluntarily donate to support society to meet some of its pervading challenges in one way or the other. There seems to be an urgent need among enterprises across the globe to hold fast to corporate social responsibility (CSR) as a synergistic platform to enhance corporate offer and competitiveness [1]. From a historical point of view, more than a century of immense corporate fecundity has formed the ecological conditions, shaped the current understanding of CSR, and made a profound impact on CSR research and practice across the globe [2]. Along this CSR evolutionary trajectory, different revolutionary occurrences of historical significance have serenaded the principles, theories, practices, mechanisms, approaches, driving dynamics and stratagems of corporate social responsibility [3].

The early theories of corporate social responsibility (CSR) advocacy that emerged in the 19th century provided conflicting evidence as to why a firm should support CSR or not. In his ‘magnum opus’ “the wealth of nations”, renowned Scottish philosopher Adam Smith explained that consumers were “social sentinels” and must only support enterprises that are socially responsible. These are enterprises whose actions and inactions advance their interest without compromising the interest of the society [4]. According to Adam Smith as cited in Hedblom et al. [4], given the opportunity, an industry player will always pursue a selfish reason to satisfy its personal benefit at the expense of society. Smith believed that consumers are the best stakeholders to guard the welfare of society by ensuring that only goods and services of social companies are patronized.

However, there are many dissenting voices to this notion of corporate social responsibility. For example, Milton Friedman believed that business organizations are established just to satisfy the profit motives of their shareholders [4,5]. With time, more convincing CSR theories (e.g., social contract theory, stakeholder theory, etc.) have emerged and the field has so matured beyond being a simple corporate sidebar.

Several contemporary enterprises have gained a better appreciation of the need to develop a corporate conscience and stimulate socially responsible activities. This is the only way by which they can obtain social legitimacy and bolster brand value to safeguard its continuous existence and prosperity [6,7]. CSR has become a business strategy that is well established not only in the academic literature but also in practice.

Long before the industrial revolution and many years afterwards, several public and global health crises have driven and continue to drive changes in society and the workplace. Health crises are harder to understand as they are typically infrequent and unpredictable. Thus, health crises are akin to the black swan as they are unexpected yet have severe consequences. Since December 2019, the enormity of the impact of COVID-19 on corporate organizations and the global economy has triggered an unprecedented and unfathomable shift in corporate social responsibility paradigm and practices as the world battles to contain the corona virus [8]. Yet, long before COVID-19, the discombobulating effect of the Spanish flu, cholera, malaria, HIV-AIDS, environmental health crisis, H1N1, MES, ebola, obesity, and the opioid epidemics, etc., on corporate stability had catalyzed the incubation of public-health-led CSR strategies to advance the frontiers of corporate social responsibility.

Even though general literature on health-related CSR is dotted across different extant studies, a synthesis of how public health crises have shaped the past, present, and future of CSR is limited. Moreover, not every health pandemic has become an extremely topical issue to influence the cause of corporate social responsibility. This is because only a few of such epidemics manage to gain the attention of the global public, international organizations, and multinational corporations to elicit their interest, advocacy, and support. This is because the corporate world is profit oriented and only global health crises that have the potential to cause wide range disruptions in business or improve business interest of firms often get corporate social support [8]. This review explores the global health crises that have influenced the evolution of CSR practices and principles and how knowledge of them can help explain the potential impact of COVID-19 on CSR. Consequently, the following are the research questions this study seeks to answer:

(a) To compare how different global health crises influenced the evolution of corporate social responsibility practice.
(b) Whether business organizations are able to balance economic intentions and the need to support societal through CSR programs during global health crises.

The rest of the article is structured as follows. The Materials and Methods section is explained after this introductory section. Next, we discuss the findings from literature under five main topics. The conclusions and theoretical implications of the study are drawn. The limitations and future research directions are then outlined to conclude the paper.

## 2. Materials and Methods

**Figure 1:**
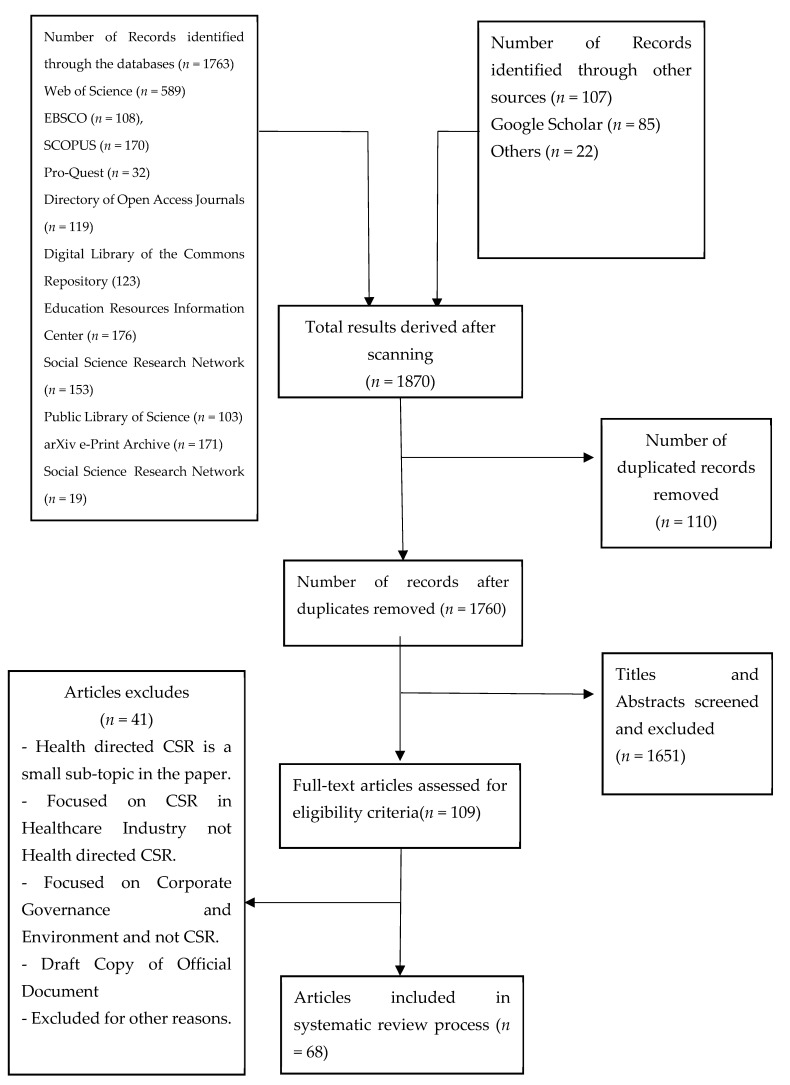
PRISMA model.

The effect of global health on CSR is a complex issue but the debilitating effect of COVID-19 demands timely and accurate information to support enterprises. As such, an integrative review method was chosen but ideas were borrowed from the Preferred Reporting Items for Systematic reviews and Meta-Analyses (PRISMA) checklist to ensure that the reviewed scientific literature was not selected arbitrarily. Figure 1 shows the graphical representation of the selected activities. Consistent with the prior works of D’Aprile and Mannarini [9], corporate social responsibility was treated as a multidimensional construct and its mechanisms, processes, and evolution are driven by an ensemble of sophisticated intrinsic and extrinsic factors. These factors sometimes come closer and move apart. In other words, the context of CSR motivation and practice, theoretical expositions and assumptions, policy and regulatory framework are shaped by a matrix of socio-cultural and economic factors that evolves overtime. This makes CSR practice a dynamic and constantly and rapidly evolving endeavor for business organizations that wants to take advantage of its benefits. To this end, this research synthesized and evaluated the most current studies that highlight how health-related factors have shaped contemporary CSR practices and its future trend in the midst of COVID-19. Health drivers of corporate social responsibility were first extracted from available studies and clustered in accordance with evolution, purposes, diffusion into CSR practices, and effect of such diffusions.

### 2.1. Search Strategy

A total of 10 bibliographic databases were shortlisted for extended search based on initial screening on related contents between February 2019 and February 2021. This date was chosen to give enough time to understand the impact of the COVID-19 pandemic on enterprises after it emerged in December 2019. The databases were the Web of Science, EBSCO, SCOPUS, Pro-Quest, Directory of Open Access Journals, Digital Library of the Commons Repository, Education Resources Information Center, Social Science Research Network, Public Library of Science, and Social Science Research Network. These databases were chosen because of their credibility, volume of information they store, and impact factor of articles stored in them regarding CSR and public health. Even till today, there are several aspects of COVID-1 that remain unclear and research, knowledge about its impact on corporate practices is still developing. As such, most of the available studies are still deposited in pre-print databases awaiting peer review. For this reason, frequently cited pre-print databases such as arXiv e-Print Archive were consulted for additional information. For each database, distinct and hierarchical search cluster terms were defined i.e., main topic, subtopic, and specific theme.

Narrative search was used to select the articles. The search terms (public health interventions, health-related CSR, environmental health-related CSR, COVID-19-related CSR) were combined through Boolean operators such as AND/OR. The search terms were entered individually in English. Truncations as well as wildcard characters helped to improve the sensitivity and precision of the searches. The initial searches did not discriminate in terms of publication time frame, research design (qualitative/quantitative research, primary/secondary research), peer review criteria (essay or dissertation or academic paper). The initial search yielded 1763 articles and was supplemented with additional hand searches in Google Scholar and a cross-check of the reference lists of studies included for analysis. Through this process, 107 additional articles were retrieved and added to the selection process.

Table 1 presents the summary of the different types of global health crises that are of CSR concerned initially extracted from the articles in the databases and other sources. The difference in the number of cases per source and the total number of articles from each source stems from the fact that the cases overlapped across the articles. In other words, in some instances, a single article discussed more than a single global health issue that has affected CSR practice. Most importantly, three groups of global health epidemics emerged as dominantly discussed in the extant literature and they form the basis for the discussion in this paper. For example, in the initial search, environmental health crisis was reported 1316 times across the articles whereas the influence of HIV-AIDS on CSR was reported 1253 times in the studies. On the other hand, COVID-19 was reported 298 times whiles the opioid and obesity epidemics are represented in 283 and 163 studies, respectively.

### 2.2. Screening

The articles were initially screened to remove duplicates in a two-step process. The entire list of articles was imported to four citation managers namely Mendeley, EndNote, Sciwheel, and Zotero. Four well-trained research assistants with expertise in library and archival reference management information system removed all duplications. This was strictly supervised by the author. The screened results from each of the four citation managers were carefully compared. After manually inspecting and validating the articles, the author compiled the final list of qualified articles. From this process, a total of 110 duplicate articles were removed from the list of 1870 articles, leaving a total of 1760 qualifying articles. These final articles were further validated by the author and the research assistants.

### 2.3. Eligibility

A strict eligibility criterion was used to determine qualifying articles for the final review. Firstly, the article should be available in English language. Secondly, the article must focus on healthcare issues in corporate social responsibility including any domain or topic-specific health-driven CSR studies. Thirdly, the article should be a peer reviewed academic paper. Where the paper is not a peer review paper, then it must be a document from a highly rated, international team or recognized professional group. Official CSR documents released by multinational enterprises, international organizations such as the United Nations, International Labour Organization, etc., and papers that offer insight into the historical evolution or unique information and context for conceptualizing and theorizing health-related CSR were included. Another criterion for inclusion and exclusion was that the selected article must document available health-related CSR practices, strategies, systems, corporate initiatives, successes, failures, and future changes.

Finally, a recent article that synthesizes CSR and COVID-19 was highly recommended. Articles published in relation to corporate responsibility and the obesity epidemic, CSR and Internet addiction, opioid addiction and CSR, which are not known contagious pathogenic health crisis but have been linked with CSR in the past, were included for analysis. Whether articles were included for full-text analysis was determined by the author with the assistance of trained literature search specialists depending on whether the articles fitted well with the eligibility criteria. Publications that were disputable were further validated through a snowballing of other relevant considerations and deliberations among the research team members until consensus was reached to accept or reject its inclusion.

### 2.4. Data Extraction and Analysis

A final set of 68 articles that summarized the major public health crisis that influences CSR were selected for full-text analysis based on the following reasons. Twenty-one of them contained information on HIV-AIDS and corporate social responsibility, while 26 of them contained information on environmental health catastrophe and corporate governance. Sixteen of them described the interplay between COVID-19 and corporate governance. Other studies that directly addressed the three shortlisted subjects were included in view of their current position on COVID-19 and the new insight they provide for the future of corporate social responsibility after COVID-19. All the sixty-eight articles were qualitatively evaluated and synthesized through a four-step inductive content analysis process. In the first place, the eligible articles were scanned definitions and conceptual models that were directly developed for the target group or adapted to it or included relevant perspectives on health literature as a whole.

Next, the definitions and models were coded and extracted by the research team based on an inductive approach. Definitions and models that overlapped from the same research groups were included on a single occasion. For non-related articles that explain the same health literature definitions or models, only the original reference was added and marked accordingly. In the third stage, important background data were declined and extracted into a matrix. Some of these data include age of target group, reason for studying the target group, whether the perspective of the target group was considered in developing the definition or model or in applicability and relevance of these and the settings for which they were developed. Finally, the articles’ research design and methodological quality were assessed. Finally, the identified themes and dimensions were discussed with a whole research team in April 2020 and the feedback was integrated into the final analysis.

## 3. Results

The study selection flow diagram is illustrated in Figure 1. It summarizes the number of the studies recorded at each stage of the process. For example, the figure reveals that the initial search yielded 1760 potentially relevant citations and after screening abstract and titles, 109 were kept. Further screening of the citations led to the final set of 45 articles which have been presented for extended analysis in this report.

### 3.1. Study Characteristics

Table 2 presents the characteristics of the 68 shortlisted studies. About 27% of the results were focused on HIV-AIDs whereas 34% were focused largely on environmental health. Overlapping studies were also recorded. For example, 9% of the studies involved COVID-19 while 17% involved COVID-19 and HIV-AIDS. Further, 13% involved COVID-19 and environmental health while 11% involved environmental health and HIV-AIDS. In addition, 21% of the studies were primary qualitative research whereas 42% were secondary qualitative research. Further, 17% of the studies were primary quantitative research while 20% quantitative research studies. The settings of the study were widely variable. Additionally, 48.7% of the studies focused on the Sub-Saharan Africa while 19.3% focused on Europe. Further, 15% of the studies focused on South America and 9% were focused on the United States of America. The total number of studies that focused on Asia was 17% while focused on a global scale.

### 3.2. Study Quality

To evaluate the quality of the studies, the Mixed Methods Appraisal Tool (MMAT) was applied as shown in Table 3. Pluye and Hong [10] explain that the MMAT tool helps to provide quality appraisal for quantitative, qualitative, and mixed methods to be included in systematic reviews. The score of the MMAR results in this case is presented in Table 3. As disclosed, scores for the selected studies ranged between 25% and 83%. In addition, 4% of the studies received 25% rating based on the MMAT criteria whereas 5% of the studies received 33.3%. Similarly, 13.5% studies received 50% while 38.5% received between 60 and 80%. The remainder of the studies received in excess of 80% on the MMAT tool.

The most frequent weaknesses related to lack of discussion on the reason for studying specific organizations, the influence of the organization on the research, and researcher influence in qualitative and mixed methods studies. There were also issues with lack of a clear description of the sampling process of respondents adopted by authors in quantitative studies and sub threshold rates for acceptable response or follow-up in non-randomized quantitative studies were also recorded as major weaknesses of the quantitative research. Most of the studies had support from funding agencies or organizations for whom the research outcome serves their interest. Thus, the influence of such organizations in the conduct of the research was not disclosed by the researchers.

### 3.3. Differences and Similarities between the Nature and Effect of COVID-19 and HIV-AIDS

Table 4 presents a comparative analysis of some of the key characteristics of the main epidemics that have influenced corporate social responsibility within the last couple of years. Ten main epidemics were noted from the extant literature. These were COVID-19, Spanish flu, HIV-AIDS, cholera, environmental pollution, malaria, MES, ebola, opioid, and obesity. Predictably, COVID-19 frequented in the studies most as a target for CSR. This was followed by HIV-AIDS and environmental health. The opioid and obesity pandemics have received the lowest concentration of CSR articles about them. These ten different epidemics have affected CSR in its current practice because they have attracted the attention of the international community and agencies such as the United Nations as well as multinational organizations. There were differences that were found among the ten epidemics and these differences and similarities equally affect CSR practice and even the enormity of commitment that is invested by companies on their related CSR [11,12]. The epidemics differ in terms of the scope of geographical coverage of infection. For example, COVID-19 is global but MES was restricted to the Middle East and parts of Asia. Even though malaria and cholera are global, most of the studies emerged from developing countries in Sub-Saharan Africa, South East Asia, South America, etc., and these are the places where most of the CSR activities are concentrated.

A similar observation was made about HIV-AIDS. It is a global pandemic but southern parts of Africa have received the highest concentration of studies and CSR resources from countries. The seasonal variation in the infection is also one of the sources of differences. In this case, COVID-19 and MES vary according to weather conditions while most of the others do not. The scale of public panic over COVID-19 has been enormous compared to the panic that greeted HIV-AIDS and MES [13,14,15]. In the case of COVID-19, MES, and the Spanish flu, the studies show that physical lockdowns were used to control infections. Together with cholera, isolations were also used to control infection rate. Even though HIV-AIDS had its own stigma, the scale of COVID-19 and Ebola were enormous. We found that obesity also belongs to this category of epidemics with some stigma. With ebola, MES, COVID-19, cholera, and the Spanish flu, mass gatherings were major sources of infections but there is the possibility of early detection and treatment for all the diseases with the exception of HIV-AIDS which can be moderated but not treated [16].

Other considerations that distinguish these epidemics include effect of underlining conditions of criticality of illness, age variation in infection rate, gender variation in infection rate, geographical concentration of highest rate of infection/deaths, and scale of frontline deaths. The rest of the differences and similarities include the scale of direct impact of epidemic on socio-economic activities globally, scale of direct impact of epidemic on cost to businesses globally, scale of direct impact of epidemic on business revenue globally, and scale of use of inter-government regulations to control infection [17]. Finally, differences and similarities also exist among the ten epidemics in terms of how the epidemic disruptions, cross border lockdowns to prevent spread of epidemics, rate of infection among people, mode of transmission of epidemic, intensity of CSR, and criticisms of CSR.

## 4. Discussion

### 4.1. Business Interest versus CRS in Response to the Spanish Flu

Reviewing the paper by Ntim [18], they contend that business organizations often claim that the main reason why they are involved in CSR is to support the society to overcome some of its critical challenges. However, the authors note that business organizations are driven into corporate social responsibility because it is an opportunity to minimize cost or improve revenue and less of an opportunity to support society. It is therefore important to look at some of the losses that are occassioned by epidemic outbreaks. According to Bolton [19], businesses began to support the fight against epidemics only after they estimate how much loss they can realistically avoid or how much revenue they can maximize. The events before corporate involvement in CSR during the 1919 Spanish flu is examined in Mahajan et al. [20]. The Spanish flu is imporant in this context because it closely compares to COVID-19 in terms of infections (500 million) and mortality (50 million) and its impact on macro and micro economic indicators that determines the survivability of business enterprises. This flu occurred at a time when the global economic system was emerging from the ashes of the WW1.

According to the Federal Reserve Bank of St. Louis and the Arkansas Gazette, popular merchants in Little Rock (Arkansas) reported 40–70% decline in business revenue and other retailers lost nearly two-thirds of their income. Despite the increase in the sales of drugs, bed, springs, and mattresses, thousands of irrecoverable goods were lost daily due to poor storage system or lack of it [21]. The negative effect of this influenza on businesses in Memphis (Tennessee) was even worse. The banks were unwilling to offer them overdrafts due to panic withdrawal. The Memphis Street Railway and the Cumberland Telephone Company redeployed half of their employees and that lead to a cut in production and services. On the 18th of October 1918, the “Tennessee Coal Mines” shut down its main operations unit causing fifty percent decline revenue over six months. Sanyal [22] and Welker [23] also reports that several mines throughout east Tennessee and southern Kentucky were closed down. The coalfield in Tennessee, which was one of the largest coal production hubs, had only 2% of its 500 employees available to work.

Typically, small and medium scale enterprises were the worst affected by the Spanish flu just as it is the case with COVID-19 as the latter were unable to keep up with some manufacturing schedule or to delayed trading until the market conditions improved. The worsening macro-economic indicators also laid a strong foundation for companies to support the fight to end the Spanish flu. Delmas et al. [24] reveal that the macroeconomic environment under the Spanish flu equally plummeted. In the US alone, the death toll led to a sharp decline in GDP by 1.5% and consumption by 2.1%. Both large and small scale businesses were affected by the sharp rise in inflation by 5% and interest rates by 13% by the end of the first six-months of the pandemic. It is worth noting that these indicators were already at their terrible levels due to WWI. Shaukat [25] also explains that by the time business organizations saw the need to be involved in halting the continuous spread of the Spanish flu, the stock returns had dropped by 7% and the safest government bonds had tumbled by 3.5%.

The trend in the US was not an isolated case because the entire global economic indicators were heading towards danger to the detriment of international merchants and cross border trade which was already under siege by the aftermath of WWI. Without prejudice to the growth in pharmaceutical, medical supplies, and healthcare, Barro et al. [26] explain that the Spanish flu reduced the global real GDP per capita by nearly 6%. Similarly, Correia et al. [27] report of an 18% decline in the US’s manufacturing output. In Sydney, the sales volume fell between 25% and 40% while several hitherto large retailers folded up as a result of decline in foot traffic.

Faced with such challenges, business organizations had no option than to be involved in what they called CSR. Initially, large scale business merchants supported government through information dissemination and increased support to their affected employees. With time, a number of flourishing merchants opted to help pre-finance the manufacturing of vaccines in return for preferential trade treatments that had been rolled out by the governments. As the disease was subsiding, employment became one of the major CSR tools that companies rolled out to the extent that it was economically beneficial [28].

Idowu et al. [29] report that the aftermath of WWI had also created a large stock of veterans that were struggling to reintegrate into the society. Several merchants and larger corporations took advantage of the opportunities to reabsorb veterans into the labor market in return for government stimulus packages (tax exemptions, wage subsidy, rent subsidy, preferential supply contracts, special export and import licenses, etc.) were targeted at companies that could help solve some of the political problems created from WWI. In order to take advantage of government stimulus packages, large manufacturing companies re-absorbed returning veterans into the labor market as a form of corporate social responsibility. Malovics et al. [30] report that since the economic benefit that were to accrue to the enterprises inspired their decision to engage in this form of CSR, the selection of veterans also came with challenges. This is because business competed for veterans with professional training and physical capabilities that were suitable for their business operations to the detriment of veterans who suffered restraining disabilities during WWI.

### 4.2. Business Interest versus CRS in Responding to Malaria and Cholera Outbreaks

According to Reinhart and Stavins [31], an analysis of how companies responded to CSR with the onset of malaria and cholera pandemics indicates that these were also mostly inspired by the need to boost business revenue and minimize business cost. According to the World Economic Forum, malaria is bad for business. When the malaria crisis first emerged, several corporate employees unknowingly became agents for community transmission and this highly affected their businesses and the local economy [32].

The local economies lost due to deteriorated human capital, losses in savings, and investments and loss of tax revenues. In a survey conducted among companies in Sub-Saharan Africa, Central Asia, and South East Asia, the World Economic Forum reported that 72% of companies had suffered revenue losses and high cost as a result of malaria-led employee absenteeism, reduced production and productivity, and escalating benefit cost [33].

Overseas, UK businesses such as BHP Billiton, which owned Mozal Aluminum Smelter in Mozambique, lost 7000 employees and 13 expatriates’ deaths in two years. The UK-based mining and metals company had investment of over US $1.4 billion at that time but the state of the malaria outbreak over two years made it difficult to recoup their investments. The company spent an estimated US $2.7 million to help control malaria-related illness, absenteeism, and treatment before the company resumed uninterrupted business. Even though these commitments are classified as corporate social responsibility intended to support society to meet its health crisis, Alvarado-Herrera et al. [34] argue that it was only through investment in the health of their workers that they could be guaranteed uninterrupted production cycle, minimize cost, and boost competitiveness.

This supports the work of Schönherr et al. [35] that it is through CSR for malaria that business organizations can scale back malaria to reduce malaria-related illness, deaths, expenditure, absenteeism, and even loss of staff. In some of the worst affected cholera countries in the world such as Zambia, Mozambique, Ghana, etc., companies often use their resources and infrastructure to secure external funding, scale up interventions which may have taken a long time to come, but being a good corporate citizen in a time of epidemic is the only way to strengthen business reputation and obtain self-perpetuating social legitimacy for the future [36].

For example, the M2030 was introduced by the Asia Pacific Leaders Malaria Alliance (APLMA) as a forum to harness and inspire CSR among concerned enterprises. Its stated objective was to unite businesses, consumers, and health organizations towards the elimination of malaria in Asia and Pacific. Within a year of commencement of operations, many companies became attracted to the M2030 and the idea to support the funding of malaria programs [37].

However, a lot of companies also enrolled into the program because M2030 as an inter-governmental initiative, permitted partner companies to use the M2030 brand for campaigns, and even rebrand some of its products and services to enhance their sales. Many of the companies therefore saw this opportunity more as a cause-related marketing strategy instead of a humanitarian gesture to society, hence the rush to enroll in this coordinated CSR program [38]. Not surprisingly, companies that did not see much economic benefit from the M2030 later became dormant members. Most of the critics of the M2030 CSR project still believe that it is avenue for the benefit of businesses to boost their corporate reputation. A recent concern by Chaung and Huang [39] gives credence to this factor when they assert that the success of the M2030 project and the continuous support from firms is contingent on the M2030 brands ability to help drive sales and customer retention.

The cholera outbreak is one of the major epidemics that has always attracted CSR activities due to its effect on business cost and business revenue. Marco-Fondevila et al. [40] explain that cost and revenue considerations have persistently informed business intentions to engage CSR right from the outbreak of the Asiatic “cholera” which broke out in 1832. According to Alon et al. [41], the Asiatic cholera was believed to have originated from India but moved westwards through Eurasia, Europe, and eventually the United States with citizens put on edge. Predictably, citizens along cities where the pandemic arrived left the city in haste as doctors pressed for public announcements to alert households of the debilitating effect of the ranging pandemic.

Significantly, Albuquerque [42] asserts that public health boards and mayors were initially hesitant to release timely information due in part to the influence of large business organizations, prominent bankers, and merchants that had bankrolled the politicians into office for fear of loss of business, revenues, trade deals, and excessively huge cost as a result of the panic. It is documented in Ozil and Arun [43] that even when the pandemic was at its highest peak, hotels wrote to local newspapers to run notices that their premises were free of cholera and open to business in disregard of public health recommendations. “The American Hotel,” the Evening Post dutifully reported, “neither has been nor will be closed.” Yet, as the fear eventually unfolded, CSR was used as the key strategy to recoup losses. Besides the drying up of merchandize, the epidemic changed even the personal lifestyle of the rich merchants and their spouses had to bake breads themselves due to the closure of city shops [44]. The famous Pearl Street goods market and city dwellers withdrew their savings to the chagrin of banks that had run out of liquidity.

More recent outbreaks of cholera amidst business losses and CSR interventions further supports the idea that business organizations are more focused on the benefits derived from them [45]. For example, in 2017, MTN Zambia spent more than half a million Kwacha to procure 300 bins, 60 vests, towels, hand sanitizers, soaps, and other equipment to help curb the cholera outbreak in Lusaka. Even though all of these materials were bought on the open market, a lot of money was spent to rebrand them with MTN symbols and promotional messages.

A news commentary in Gentilini [46] criticized MTN for commercializing the epidemic situation extensive promotion of MNT through a non-commensurable donation. This reaction was due to the fact that within two months, the whole country had been painted with the yellow MNT drums and symbols, drawing public criticisms, earning them free publicity that would have cost them more money than the cost of the donation if they had paid for the public spaces. One newspaper sarcastically reported that the outbreak of the yellow epidemic (in reference to MNT colors) was more than the cholera outbreak.

The Zimnat Group did the same thing when cholera broke out in Zimbabwe in September 2018 [47]. Over three months, the company donated 20,000 L of water to several locations including Budirio 5D Current Shopping Centre in Harare in tanks that were hilariously branded in the company’s green colors to boost their prominence during the epidemic time. The intimidating green presence across the length and breadth of the capital city attracted several criticisms from the press for attempting to commercialize the pandemic.

Another way CSR comes under the cover of business promotion during an epidemic is how Kia Motors and LG Electronics (both Korean owned companies) have partnered Korea-based International Vaccine Institute (IVI) to provide an emergency cholera vaccination program in Malawi and Ethiopia, respectively [48]. On the face of it, these multinational giants seek to improve quality of life through such interventions without disclosing the business opportunities it creates for them. However, the IVI is a United Nations initiative based in Korea and stimulates partnership with companies for humanitarian purposes by offering them several incentives to promote their businesses. For their support for the vaccine program, KIA and LG Electronics benefit through access to high global networking of UN agencies and governments [49]. They also get support in the form of connectivity with stakeholders, its tools, resources and trainings, local network support in 85 countries and more importantly, the moral authority, knowledge, and experience of the United Nations which can facilitate access to major international and national level contracts.

### 4.3. Business Interest versus CSR Response to HIV-AIDS

In Vaccaro et al. [50], it is reported that support for private sector (NGOs) involvement in the fight against HIV-AIDS was started by non-business groups such as Family Health International. These initial efforts were not classified as CSR since they did not emanate from corporate enterprises. Due to lack of funding, most of these non-business organizations focused their HIV-AIDS intervention on data collection and analysis, individual risk assessment, prevention and cure education, impact assessment of HIV/AIDS on specific industries, and development of proposals to guide workplace prevention and care. Frequent engagement with these NGOs stimulated corporate interest to start their own programs through CSR [51].

Just as it is with other forms of public health concerns, some critics of HIV-AIDS-related CSR believe that business organizations use the epidemic platform to consolidate their importance, minimize cost, and optimize potential business opportunities created by the epidemic. According to Ferreira [52], several business opportunities are inherent in the pandemics that enterprises can explore, and HIV-AIDS is one of those with high business interest and this can be procured through CSR. The interest of MNCs in HIV-AIDS-related CSR, emerged crystalized as the global advocacy for HIV-AIDS intensified in the early 1980s. At this point in time, the pandemic was largely concentrated in Sub-Saharan Africa [53] before growing to every part of the world.

The early signals of the catastrophic effect of HIV-AIDS to disrupt a wide range of socio-economic and corporate activities notwithstanding, serious documentation of HIV-AID-related CSR programs started in the late 1990s [54]. At this point in history, the ominous or devastating effect of the pandemic on human resources and economic development had become entrenched across continents [55]. With exponential increase in the number of infected persons across industries and countries, a global alarm was sounded by the International Labour Organization (ILO) in 1990, to highlight the epidemiological influence of HIV-AIDS on individuals, households, workforce, employers, and organizations [56].

The ILO subsequently engaged several enterprises to begin incorporating appropriate strategies to control the threat posed by the HIV-AIDS pandemic to decent work, productivity, and national development [57]. As documented by the World Health Organisation [58], this initial effort formed the basic documentary framework for discussions at the Special High-Level Meeting on HIV/AIDS and the World of Work in Geneva in 2000. To gain a deeper attention of business enterprises to invest their resources into HIV-AIDS-related CSR, the International Labour Organization (ILO) and the United National Development Program (UNDP) released a joint document that provided statistical evidence of how business were going to suffer (due to labor shortages) if the pandemic persist [59]. Classified data from thirteen African countries, Thailand, and Haiti collected from the United Nations Population Division were analyzed with the e ILO-POPILO software [60]. Based on the ILO assessment, business became conscious of the fact that rapid increase in HIV-AIDS infection among the 20–49 years bracket was significantly altering the age and sex distribution of the labor force and that could affect enterprise production [61].

Three main problems were identified by enterprises of this persistence, hence the need to join the fight against HIV-AIDS through CSR. Firstly, high HIV-AIDS-related deaths were pushing children or less experienced people into the labor force. Secondly, experienced employees with HIV-AIDS withdrew from the labor force early and thirdly elderly people had to be retained in the labor force due to rising economic dependency due to the early death of younger employees [62].

In 2001, 17 eminent and visionary companies founded the Global Business Council on HIV/AIDS. In the work of Harvey [63], this initiative added the needed global impetus to place HIV-AIDS at the center of corporate solidarity and responsibility. Harvey [63] again posits that the Global Business Council on HIV-AIDS collaborated with the UNDP, the Prince of Wales Business Leaders Forum, and Nelson Mandela Foundation to develop a business response to confront HIV-AIDS head-on in mainly developing countries [63]. The Global Business Council (GBC) developed a broad range of CSR strategies i.e., public information strategies for its members and others. It also set up the annual award for business excellence to recognize the contribution of businesses to the HIV-AIDS pandemic. In 2002, the Global Business Council participated in the UN General Assembly Special Session on AIDS (UNGASS). It used the forum to expand the need for high level business response to HIV-AIDS among prominent business leaders and international policy-makers. Its frequent publications on HIV-AIDS and other health-related crisis continue to inspire new business responses to global health crisis including the HIV/AIDS pandemic [64].

A major success of the GBC is that 46% of businesses in the US got involved in some kind of HIV/AIDS philanthropy across the globe. However, critics of the Global Business Council on HIV, which has since changed to the GBCHealth, believe that the companies have benefited from HIV-AIDS more than the communities. Firstly, the decision to appoint the then World Bank president James Wolfenson as the chairperson of the club of companies instead of a technical person with practical experience in humanitarian issues was the first indication that the interest was about creating business opportunities through HIV-AIDS-related CSR other than supporting society [65].

When the club was formed, it developed 6 key approaches to work but critics argue that five of them are just focused on the benefits that the businesses will extract from their undertaking rather than the support for communities and affected individuals. The first priority of the group was to convene and connect businesses, governments, multilaterals, and civil society while the second is to represents businesses in driving the creation of high-impact partnerships-business-to-business and business-to-government [66]. The third goal of the group is to provide recognition and visibility to companies while the fourth is to represent business in key global health settings. The last objective stated by the group is to provide guidance to companies on their workplace and corporate social investment initiatives. Faced with these objectives, and guiding principles, it becomes difficult for one to clearly accept the previously held notion that patients and affected communities are more important in the quest to fight HIV-AIDS than the personal interest of the companies. The analyzed studies also present specific MNC interventions in HIV-AIDS through corporate social responsibility but the twin-face of an organization as using such platforms to promote their interest is also evidently shown [67].

To support the claim that businesses that get involved with HIV-AID-related CSR were more interested in protecting their interest rather than society, Makwara et al. [67] again highlight the case of Daimler Chrysler, De Beers, Nestle, Johnson and Johnson, Coca-Cola and Unilever, Proctor and Gamble who were among the companies that first started HIV-AIDS-related CSR in Kenya and South Africa [68]. These companies only conducted research on the association between HIV-AID- related CSR (prevention and treatment of HIV-AIDS) and the company’s balance sheet [69]. These research studies confirmed the need to be involved in HIV-AIDS related CSR in order to protect the firm’s greatest resources (human resources). Nestle went further to simulate how employee work productivity differs between infected employees with company supported medication and those without company supported medication.

These companies were alarmed by the potential high level of absenteeism, frequent sick leave, poor organizational citizenship behaviors, and even death that permanently terminates the work relationship. The companies understood the potential loss of revenue, customers, and the high cost that they were confronted with if the situation should persist and solicited the support of the media to help eradicate HIV-AIDS [70].

Significantly, the media accepted the partnership from major companies because they benefited from the publicity and promotional budgets of the corporate enterprises. With this knowledge, several individual organizations began to navigate company specific approaches and mechanisms to incorporate HIV-AIDS programs into its corporate social responsibility budget to support affected employees, community, and country.

Adegbite et al. [71] reechoes this when they say that these initial CSR initiatives to manage the threat of HIV-AIDS in the early part of the 1990s were largely focused on how companies could protect their employees from acquiring the HIV-AIDS virus and prevent avoidable intra-organizational spread of the pandemic

Even though HIV-AIDS-related CSR entered a different phase in the late 1990s, the effect of business interest over societal interest increased [72]. For instance, Coca-Cola partnered with UNAIDS to provide extraordinary support against the HIV/AIDS fight in Africa [73]. This collaboration was the first and largest private sector initiative of a major global brand to implement a systematic philanthropic and corporate citizenship program with a specific focus on HIV-AID in Africa [74]. This initiative allowed Coca-Cola to focus beyond the employees living with AIDs and bring the larger community in focus, support infrastructure to support patients, use its wide range distribution channels to market HIV-AIDS related resources, while strengthening its human resource policies to ensure greater involvement in the fight against HIV/AIDS [75]. However, this massive involvement of Coca-Cola in HIV-AIDS came at a time when the company was facing severe backlash in South Africa and other parts of the content.

It is recalled that in 1982, black workers led a boycott of Coca-Cola products in protest against low wages, pension funds, and the depleted bargaining power of workers union. Since then, several critics have referred to Coca-Cola as a conduit of economic support for white South Africa and its apartheid system. International friends of South Africa such as Tennessee State, Penn State, and Compton College in California, even established a “Coke Free Campus” while the Georgia Coalition led a series of protests to move Coca-Cola out of South Africa [76]. At that time, Coca-Cola rolled out massive support to improve housing and education for black South Africans and sell 30% of shares in bottler and 50% of canning operations to native South Africans but these were rejected. It was within this time that the HIV-AIDS prevalence rate escalated in Southern Africa and Coca-Cola seized the opportunity of CSR to hold on to its stay in South Africa [77].

Again, in 2015, it came to light that Coca-Cola had influenced research by the Global Energy Balance Network to promote research findings that blamed obesity on lack of exercises and not on reducing the intake of calories. This was deliberately engineered to deceive the public of the impact of the excessive sugar content of Coca-Cola in the spread of obesity and type 2 diabetes. This happened at the time Coca-Cola had just announced major humanitarian support for a series of health crises across the globe [78].

The CSR effort of the Corporate Council on Africa (CCA) is also addressed by Kurland [79]. Corporate Council on Africa (CCA) is a leading business association of American enterprises that connects business interest in Africa. The group formed two lobby groups i.e., a Task Force on AIDS in Africa and the Coalition for AIDS Relief in Africa that brings together major pharmaceutical companies, such as Abbott Laboratories, Bristol-Myers Squibb, Pfizer, etc., to lobby Congress on how the President’s Emergency Plan for AIDS Relief (PEPFAR funding) can benefit business interests in Africa. Since its inception, the Corporate Council on Africa has released periodic timely reports to support concerned enterprises to standardize their HIV-AIDS-related corporate social responsibility programs.

The main advantage of the mode of operation of the Corporate Council on Africa is that it partners high profile companies including Ford Motors, Coca-Cola, Boeing, Microsoft, etc. to work through local groups and governments to design, develop, and implement culturally sensitive strategies to combat HIV/AIDS among the African workforce [80]. The CCA also has its critics on the genuineness of the humanitarian endeavors that it engages it. For example, Shingal [81] explains that even though the CCA considers itself a non-profit making enterprise, its main objective as describe by the council is to promote business and investment between the United States and the nations of Africa which are all profitable ventures. The first three of its key goals equally gives credence to the fact that CSR is a pathway to consolidating business interest for its members other than the society. For example, the first goal of this enterprise is to work closely with governments, multilateral groups, and businesses to improve Africa’s trade and investment climate and to raise the profile of Africa in the U.S. business community. According to the CCA, its most important goal is to support member companies to increase their investment in and trade with the nations of Africa. Thus, CSR is therefore one of the ways by which they can have access to the African market [82].

The role of the banking sector in incorporating HIV-AIDS-related programs in their CSR activities is also well documented by Nicola et al. [83]. For example, in 2003, the Standard Chartered Bank launched the “Living with HIV” project to support the global fight against the HIV-AIDS epidemic. Through this program, the bank trained staff volunteers as advocates (Living with HIV Champions) to handle HIV/AIDS-related issues within and outside the organization [84]. By 2017, the Standard Chartered Bank had provided HIV-AIDS education to more than 75,000 employees. Currently, the bank has an active HIV-AIDS community education program across the globe. This program has trained, empowered, and resourced more than 3 million individuals and organizations (particularly in Africa, Asia, and South America) to support [85].

In the work of Coussens and Harrison [86], they point out that unlike COVID-19, HIV-AIDS-related CSR in Asia did not start early, relative to the case in Africa. However, as the disease swept across Asia, corporate enterprises became aware of its debilitating effect. To this end, most notable Asian companies have also scaled up their effort to support HIV-AIDS-related programs. In India, for example, companies such as Tata Tea Ltd., Larsen & Toubro, Modicare Foundation, Aditya Birla Group, Apollo Tyres, SAIL, and Bajaj Auto etc., have been actively involved in supporting HIV-AIDS advocacy. Despite the initial set back, companies in South East Asia have many encouraging examples of public-private led CSR partnerships supporting promotional activities [87]. The main CSR activities include promoting HIV/AIDS prevention, support, and care initiatives. In the Asia Pacific region, in particular, many companies have the UNDP’s Regional HIV and Development Programme through donations and other forms of support [88].

Again, in India, the Steel Authority of India Ltd. (SAIL) started the SAIL AIDS Control Program (SACP) to create local awareness and support community advocacy programs through sponsorship [89]. It has partnered with India’s National AIDS Control Organization (NACO) and other inter-sectorial collaborations to school an AIDS education programme, family health awareness campaign, safe blood and blood products, and establish voluntary counseling and testing center.

It has also supported the annual World AIDS Day Celebrations as well as initiating exhibition and displays counseling and guidance and AIDS Art Centers. Johnson and Johnson is another important partner in the global fight against the HIV-AIDS pandemic in all forms as part of its role in attacking neglected tropical diseases (NTD). Over three decades, the company has established global partnerships in Asia and Africa [90]. To date, Johnson and Johnson has committed to HIV-AIDS partnership programs in 25 African countries (Kenya, Swaziland, Botswana, Cameroon, Zambia, Senegal, Liberia, Zimbabwe, Somalia, Malawi, Morocco, Cape Verde, DRC, South Africa, Sudan, Namibia, Mozambique, Eritrea, Tanzania, Ethiopia, Egypt, Nigeria, Ghana, Sierra Leone, Rwanda, Uganda) [91].

In these countries, it partners with different national and International NGOs to intervene in mainly HIV/AIDS anti-stigmatization advocacy and capacity building of HIV-AIDS advocacy groups and foot soldiers. Even in the case of Johnson and Johnson, these interventions are not without criticism as to being a tool to rebuild its damaged reputation. By the end of 2018, Johnson and Johnson had been implicated in 500 opioid-related cases. It is one of the companies blamed for the escalation of the opioid epidemic in the United States. Beside problems such as foreign bribery accusations, consumer fraud settlements, illegal marketing, and product recalls, J & J faced public criticisms for its role in the manufacture and sale of the cancer-causing baby powder scandals which affected its corporate reputation [92]. Faced with this crisis which directly affects human life and social goals, many critics see their effort at CSR as an attempt to clear up their battered image to remain competitive in business and not to support society as they claim to be doing.

### 4.4. Business Interest versus CSR Response to Environmental Health

The effort of private companies in solving environmental health crises through CSR is one of the often criticized efforts of corporate enterprises. This is because they are perceived to be the direct agents of environmental pollution hence and only put up CSR as a smokescreen to deceive the public into believing that they are concerned about the environment. Three selected studies indicates that the Carson’s 1962 bestseller “silent spring” was a watershed moment that brought environmental health and CSR to the fore [93]. This publication raised a new level of social consciousness among corporate enterprises and explained the inextricable linkage between pollution and public health [94]. These explanations influenced the rise of environmental advocates, some of whom had long begun navigating their own path to hold businesses accountable for the impact of their operations on the environment.

Gaylord Nelson, a junior senator from Wisconsin must also be commended for catalyzing the aspirations of earth day in 1970 which ultimately led to the establishment of the Environmental Protection Agency in the US and the subsequent enactment of several pro-environment laws [95]. These laws have protected millions of men, women, and children from diseases and death as averted the extinction of hundreds of species.

In the late 1990s, environment-led CSR became a source of competitive advantage as businesses engaged in different pro-environment activities to catch the eyes of an informed public and or avoid stringent government regulations on pollution. The United Nations used the Earth Summit in Rio de Janeiro (1992) and Johannesburg (2002) to define a comprehensive vision for sustainable and eco-friendly development. Even before the Johannesburg Summit in 2002, some corporate enterprises that participated in the World Economic Forum in 2000 had accepted to partner the UN to set up the United Nations Global Compact (UNGC) at the behest of the then Secretary General (Kofi Annan) of the United Nations [96]. The UNGC was to serve as a common vehicle to diffuse shared values and principles of sustainable development to give a human face to the global market order. In July 2000, the UNGC was launched between the UN and 24 enterprises. The UNGC began to insert human rights, social and environmental responsibility values into the corporate operations to guarantee better healthcare for global public as enterprises rapidly altered their production processes [97]. The UNGC also helped to fill the environmental governance gap of the time.

Its most significant achievement is that it defined ten principles and values to guide corporate pro-environment behavior [98]. Secondly, it formulated guidelines on the mechanisms by which the ten principles and values can be incorporated into a company’s operational strategies, working procedures and programs and policies to help create a long term corporate culture of integrity that prioritizes the health and wellbeing of society [99]. While the United Nations Global Compact was not CSR- specific tool, the ten principles it proposed played a major role in bringing social responsibility and environmental engagements to the fore of industrialization and development at the beginning of the 21st Century. The adoption of the United National Millennium Development Goals subsequent to the adopting of the Millennium Declaration in 2000 was another milestone in aligning environmental health and corporate social responsibility [100]. For fifteen years, the MDGs set the international agenda for CSR and environmental health even though it was not a CSR specific intervention project. Through the help of the UNDP, the MDG was presented to corporate enterprises as a key framework for the UN’s private sector cooperation on responsible enterprise [101]. By the end of 2015, environmental health concerns had become the most dominant health-related crisis shaping contemporary CSR across the globe.

However, critics of the UNGC believe that it used the offices of the United Nations to tacitly endorse companies that were destroying the environment while contributing huge sums of money to support the UNGC. For example, it is believed that the UNGC lacked effective monitoring and enforcement of the provisions, hence failed to hold companies accountable. On the contrary, these corporations misused their affiliation to the UNGC for public relations and economic gains under the guise of humanitarian concern. This phenomenon was christened “bluewashing” [102].

This explains why informal networks emerged to counter how corporations enrolled under the UNGC used their membership and supposed participation in philanthropic and charity-based activities of the UN as an excuse and an entry door to increase corporate influence upon international organizations. Some of these network groups that emerged to remove the veil of “bluewashed” UNGC members include the Global Compact Critics, the Alliance for a Corporate-Free UN which was led by Corpwatch, Peter Utting (deputy director of UNRISD), Maude Barlow (adviser to the President of the United Nations General Assembly), and David Andrews (adviser on Food Policy and Sustainable Development). Some leaders of Paragua’s Ayoreo tribe protested the membership of Yaguarete Porá in the UNGC. The Yaguarete Porá was a Brazilian ranching company which had illegally occupied and destroyed Ayoreo’s forests, and also concealing the presence of unknown tribesmen living in the forest. Another source of worry in the discharge of environment-led CSR is that while the environmental health crisis raged, opioid and obesity persisted contemporaneously but did not attract their attention since it provided minimal business opportunities to them [102].

Microsoft is one of the best examples of CSR with environmental health focus. The company’s CSR agenda targets the regulation of energy and water consumption, waste reduction and recycling, carbon emissions and sustainable sourcing. Microsoft also supports local communities, educates and empowers workers at Microsoft. Microsoft also provides health and wellness programs for families and other benefactors. Through the Microsoft CARES and Microsoft Ergonomics Programs, Microsoft seeks to empower and engage employees, competitors, collaborators, and the larger society to monitor and adhere environment-related CSR principles [103]. This notwithstanding, Microsoft has had its fair share of criticisms as far as the environment is concern.

The studies in relation to environmental-led CSR offer insight into the influential role of environmental factors in global advocacy and multinational enterprises decision making. According to McQueen [104], a dominant feature that has shaped contemporary corporate social responsibility since 2000 is environmental concern. Environmental concern is not an end in itself, but its consequential health effects is viewed as a form of environmental pandemic or climate change pandemic. Since the 1970s, environmental researchers have recognized that climate change, and other health stressors (both natural and man-made), can exert high influence on human health and disease in various ways but intense epidemiological reconnaissance of the crisis took time to mature.

Beyond environmental damage, the effect of climate change on health determinants such as safe drinking water, sufficient food, clean air, secure shelter, outbreak of vaccine-preventable diseases is well documented in several studies. The persistent outbreak or reports of drug-resistant pathogens and other multiple humanitarian health crises were directly traced to climate change and environmental pollution. Since the 2000s, renewed global effort has focused largely on soliciting a broad base of industrial support to transform the mechanism for tackling environmental health risks. The role of corporate institutions in actualizing this objective is the reason why environmental health promotion and prevention has become a central theme in todays’ corporate social responsibility policies [105]. However, over time, many advocacy groups including the United Nations have become disillusioned by the attempt to lower the ambition of the 2015 Paris Agreement by powerful nations with the tacit support of powerful multinationals [76]. There is the belief that environmental lethargy is growing rapidly among the top echelon of society while potential environmental led catastrophes persist. This growing pathological state of sleepiness, deep unresponsiveness, and inactivity has irked concerned citizens and environmental advocacy groups to rise up to demand greater action for the protection of the planet and its people.

According to the New South Wales State Archives & Records [106], the poignant social, cultural, and environmental advocacy of the 1970s has re-emerged as fresh and frustrated Millennials persistently refusing to settle for verbal platitudes of environmental care. Millions of such people have constituted themselves into very vibrant and sometimes violent groups who take to the streets to protest and demand a new paradigm in environmental health protection by large multinationals. Fortunately, the social and digital media have become common meeting grounds through which these discussions, protests, strikes, mobilizations, and sentiments are brought to the attention of the global audience. This has never before united concerned global citizenry and catalyzed a generation to join together to take on the environmental health challenges with the greatest possible firmness.

### 4.5. Business Interest versus CSR Response to COVID-19

A review of the selected literature again points to the fact that the next major health-related factor that can potentially shape the future of corporate social responsibility is COVID-19. Unlike HIV-AIDS and environmental health concerns, COVID-19 has gathered global advocacy within six months and its impact in the corridors of global power has been immense. This is largely because COVID-19 possesses the same if not more of the disruptive effect of HIV-AIDS and environmental health. The epidemic broke out in December 2019, as a novel corona virus in Wuhan in the Hubei province of central China [107]. At the onset, it was thought to be a domestic problem in China and its pathogenic and contagious character was not very clear even to the World Health Organization. However, overtime, the virus has spread across almost every country in the world with unfathomable momentum.

By the end of 2020, nearly 100,000,000 infections and 2,200,000 deaths had been recorded. The largest numbers of infections have occurred in the US, Brazil, India, UK, France, Spain, Germany, Russia, Canada, France, etc. In the absence of a known vaccine, political authorities in different countries have implemented several “draconian” or “non-routine” measures to break the viral chain despite the ramifying effect of such measures on economic activities and corporate stability.

For the corporate sector, some of these measures have become disruptive as they were unanticipated. The measures include stay at home orders, total lockdown of cities, closure of businesses, limits on nonessential businesses and business travels, social distances between two persons and group of persons, limit on public gatherings, closure of schools, continuous education on virus prevention measures, compulsory temperature monitoring, and quarantine of high-risk and sick persons [108]. As explained in [109], in the short term, several areas of COVID-19 are of CSR interest to corporate organizations. For example, with schools closed, companies must design working practices that enable parents to adequately spend time with their kids. They have to rank business travels to eliminate non-essential ones and provide support and for frontline workers. Enterprises must also redesign office work space to accommodate social distance requirements and reorient a new organizational culture on public health practices [110].

Even in Ghana and other less affected countries for example, the government has set up a national emergency fund that receives donations from corporate organizations. At the same time, the private sector has also set up a parallel support system under its own control to build isolation hospitals to support government initiatives. In India, the private sector has taken the responsibility to provide food support programs to worst affected by lockdowns and redeployed. This is in addition to all manners of humanitarian supports, donations in kind and in cash, transport services, food distribution, etc., for other vulnerable members of the society [111]. Direct corporate interventions in COVID-19 are well documented in the studies as well. For example, Starbucks and other telecom companies have embraced the Keep Americans Connected agenda where they are currently supporting working professionals to remain connected from remote locations [112]. The effect of COVID-19 on CSR also requires companies to guarantee financial security to the most vulnerable in the midst of business closures, reduced hours of work in response to the pandemic. A case in point is Lululemon. Despite been temporarily shut, Lululemon stores in North America indicated its willingness to continue paying employees and provide access to a pay relief fund [113]. Similarly, Microsoft has committed to paying its hourly workers their regular pay despite the dip in the demand for their services [114].

Walmart, Apple, and the Olive Garden on the other hand have updated sick-leave policies to ensure that their most vulnerable workers are adequately supported and covered. The Wall Street Journal believes that small business may suffer significant loss of business confidence as a result of COVID-19. It has therefore initiated advocacy for larger enterprises to support such SMEs through the difficult times [115]. Major enterprises such as Amazon have embraced this initiative as a form of corporate social responsibility. Amazon has set up a $5 million relief fund to support SMEs in their vicinity. Google has also pledged $1 million to support “pandemic-hit” SMEs in Mountain View, California where it operates [116]. The President, CEO, and top management personnel of United Airlines Company have decided to forego their salary to ensure uninterrupted business operations and safeguard the salaries of lower level employees. LVHM holdings has also converted a facility to quickly produce hand sanitizers for free distribution to French hospitals while Tottenham Hotspur Stadium has installed equipment to operate drive-through COVID-19 testing and swabbing for NHS staff, families, and their dependents. In this way, enterprises are creatively adapting to the pandemic to further their brand in the long run while caring for people in the current climate.

There are those who believe that business organizations involved in CSR during the COVID-19 are again employing the same mind game that characterized involvement in the United Nations Global Compact. The voluntary actions require significant outlay of resources which are non-existing when production and companies have closed down. It is believed therefore that these voluntary investments entail economic benefit for the companies and are designed to align with the objectives of investors/shareholders as profit seeking agents. For example, in North America, companies were willing to continue to pay employees when their companies were forced to close because of the benefit from the Payroll Protection Plan by the U.S. government [117]. This plan provides loans to businesses that can be forgiven (i.e., the loans can be converted to grants), hence companies do not need to repay if they maintain a certain percentage of their employees. Walmart, Apple, the Olive Garden, and Lululemon belong to this category of companies that stand the chance to benefit from the Payroll Protection Plan. Moreover, according to Verma and Gustafsson [118], the real benefit of CSR strategies are economic more than social. There are motivational benefits for employees and clients that lead to better hiring opportunities and greater market shares; cost reductions and increases in efficiency and productivity; increased competitiveness; access to sources of external financing and capital under more optimal conditions; limitation and greater control of corporate risks; and generating a reputation and long-term competitive advantages.

Gostin and Wiley [119] espouse the innate relationship between corporate social responsibility and branding. According to Zeren and Hizarci [85], how enterprises use CSR to respond to the changing phases of COVID-19 can influence their brand image which is needed in the post epidemic reconstruction of firms. Through CSR, the values of honesty, dedication, and community support can authenticate the brand value of companies in uncontrollable times. According to Daniels [120], companies that support or work with NGOs and other charities on COVID-19 can foster strong social relationships through genuine and mutually beneficial care. It offers enterprises the opportunity to build new relationships and better communication engagement.

In this case, COVID-19 can provide the CSR and business community teams with the opportunity to re-think and re-structure plans to place community needs at the top of their conversations. In some countries also, practices that qualify for CSR activities are being redefined under COVID-19 with strict guidelines. For example, the government of India has instructed that corporate contributions made towards the PM care fund will be regarded as CSR whereas contributions given to the prime ministers fund will not qualify for CSR donation. Thus, in this way, CSR contribution can significantly affect corporate tax assessment and access to other state support systems available to companies that are actively involved in one form of CSR or the other.

In the midst of this challenge, the effect of COVID-19 on the global economy poses one of the greatest threats to corporate involvement in CSR. At the end of April 2020, an estimated amount of nearly $17 trillion worth of the global business income and businesses had been wiped away by COVID-19 and $2.5 trillion was needed to reboot economies. The effect has been widespread including airlines, cruise ships, hospitality, manufacturing, and many other industries. COVID-19 has therefore assailed business organizations with unprecedented dangers of running at a loss, depleting capital retentions, inability to meet recurring debts and tax obligations, loss of an entire workforce and even customer base. Additionally, there is a strong association between capital market and public health and with the capital market roiled by COVID-19, there is the need for a revolutionary definition of corporate citizenship in this crisis time that balances voluntary support for society against the dwindling economic fortunes of corporate enterprises.

According to Bartik et al. [121], a major corporate social responsibility issue that assail enterprises under COVID-19 is navigating salary adjustments, furlough, redundancies, continuous payment of wages and salary for sick and stay at home staff, support and replacement of dead staff, and unanticipated absenteeism. Enterprises must also deal with disinfection of business offices; restructure business hours, partitioning shared office spaces among others [48]. The process of returning to a full-time work schedule has also been fraught with several challenges that have corporate social responsibility implications. In the UK, for example, the biggest trade unions are intransigent about allowing their members to work under the current conditions unless government and employers agree on a nationwide health and safety revolution to protect their members against the debilitating effect of the COVID-19 pandemic.

Alluding to the fringe interest in employer commitment to employee health and safety measures in a free market, these unions have reiterated the need for radical overhauled and stepping up of health and safety inspection and facilities at the workplace until they back the government’s effort to ease, and eventually end, the lockdown [122]. Other employee unions are equally demanding for employers to draw up and publish rejuvenated risk assessments that thoroughly clearly outline the specific measures to ensure a safe work environment for employees. Finally, there is also the demand for government to impose hefty punishment on rogue employers and state investment into more frequent health and safety inspections of workplaces.

While contributing financially and emotionally to reduce social burden of COVID-19, there is a moral obligation of enterprises to discard extreme free-market ideologies that prioritizes profit at the expense of safety of employees. The call for a radical overhaul of health and safety measures in enterprises is now urgent more than ever before. There is a moral obligation for enterprises to reform current risk assessments and cooperate more deeply with state agencies and industry collaborators to invest in the health and safety inspections at the world place [123]. Thus, with COVID-19, business organizations must recognize the need to ensure a balance between profit, people, and the planet since their economic growth depends on it. To that extent, CSR can be the game changer among various corporate strategies that creates unrivalled competitive advantage in uncontested market spaces for businesses to flourish. To this end, CSR strategies that focus on overcoming the effects of global healthcare crises must be of major concern to all enterprises because of their catastrophic effects on the very survival of firms if not nipped in the bud. Since workplace health and safety issues will not be the same after the sweeping effect of COVID-19, CSR strategies must be proactive to endure other unknown pandemics with equal capacity to disrupt business operations. These new CSR strategies must be capable of addressing the new frontiers in workplace health and safety (new normal) that have emerged [124]. There is the need for consistent research to retrace previous steps and strategies adopted to manage historical crisis of this magnitude. COVID-19-related CSR must therefore aim at alleviating mental and psychological wellbeing of its employees to embrace the wide range of changes that may occur. For example, work from home and social distancing measures vitally reduce the spread of the virus but have a negative influence of emotional wellbeing of employees [80]. Thus, leading corporations must support mental and emotional wellbeing of their staff. Moreover, navigating salary adjustments, furlough, redundancies, continuous payment of wages and salary for sick and stay at home staff, support and replacement of dead staff, and unanticipated absenteeism are just a few of the challenges that assail corporate organizations and these require a CSR response.

Companies must create innovative and regular activities to educate their stakeholders to become more committed to safeguarding future enterprise-based defense mechanism needed to diagnose, protect, treat, and rehabilitate victims and those threatened by pandemics and other emergencies that affect the stability of an organization to reduce its cost and protect revenue [83].

COVID-19 has shown that even though individual enterprise contingency plans are necessary, to fight the economic effects of pandemics, they are not sufficient by themselves. COVID-19 must teach enterprises that they operate in a complex, uncertain, and intertwined economic environment and pandemics spare no one. Companies must design CSR strategies with each other as collaborators and not necessarily competitors. This is because most of the lone ranger and disconnected CSR strategies paraded by enterprises to fight COVID-19 have not shown much resilience under COVID-19. For most companies, their existing strategies lack systematic integration and standardized performance metrics to measure their outcomes, usability efficiency, performance, and suitability which makes it difficult to determine their success or failure as effective CSR tools in the management of a pandemic [125].

Going forward, there is the need for group of firms to develop multi-agency and multi-disciplinary decision making and evaluation processes through collaborative networks. Already the idea and benefits of collaborative networks as a business strategy to mitigate the effect of catastrophic health and environmental crisis on business enterprises is gaining grounds in many different industries in the quest to respond positively to a changing business environment, and the healthcare sector is no exception. In China, for example, the biotech, medtech, and pharmaceutical clusters have accentuated effort to promote greater environmental health security through self-regulating and collaborative network of environmentally responsible behaviors, programs, and standards. These industry-led initiatives have engendered greater public support, renewed political commitment from top leaders and elicited heavy government subsidies for the industry among others. According to [126], nowadays, more and more enterprises are aware and motivated to adhere to collaborative platforms as business enablers, allowing groups of companies to improve their offer and competitiveness. This is because today’s firms must not only see themselves as competitors but as collaborators of the same goal when it comes to managing risks such as COVID-19 through shared burden. To that extent, collaborative can help groups of enterprises to develop a multi-tier system of organizations to supplement each other’s competencies to be well equipped to handle the complexities of modern healthcare issues in an innovative, efficient, and effective manner than individual firms [127].

Collaborative efforts in the fight against COVID-19 can give firms the opportunity to enjoy the vast awareness, credibility, and the brand equity that single firms find lacking in operations. Owing to their sizable budgets and greater scale of operations, collaborative firms are poised to have easier access to funds to undertake strategic programs. They will be more equipped with the necessary resources that single firms may find hard to acquire [128]. Additionally, inimitable assets like a steadfast reputation for process rigor and quality response to market opportunities might turn out to be critical for sustaining a competitive edge in crowded therapy markets. Such intangible assets could be more easily accruable to collaborative firms because of their vast portfolios and long track records of market presence and innovation [129]. These collaborative CSR strategies may lead to accumulation of different perspectives on a variety of topical issues affecting CSR practice by quickly sharing knowledge and effectively using ‘Wisdom of the corporate Crowds’. With time this, “Ideas Bank” can grow and become a warehouse with a variety of cases that can be grouped together and searched simultaneously by individual enterprises and others who need them. The next step will be to develop he mechanism to regularize the forums and develop a good publishing format and start publishing these rich case discussions, either a part of a journal or in another citable online format in public domains.

## 5. Conclusions

The objective of the study was to explore how enterprises are able to balance their business and social needs through CSR during pandemic situations. Firstly, the studies show that pandemics have similar characteristics that stimulate the business decision to get involved through CSR. Through CSR, business enterprises can strengthen societal pillars to better understand and withstand the shock of debilitating pandemics throughout history. The studies show that pandemics such as the Spanish flu, malaria, cholera, HIV-AIDS, environmental health, and COVID-19 created economic opportunities by themselves for business organizations as well. In instances where these economic opportunities were not obvious, the CSR strategies employed by business organizations were targeted at reducing the cost of the effect of the pandemics and maximizing any revenue potential. Through the various interventions to support their employees that were affected directly or indirectly by the pandemic, business organizations were actually protecting or building up their stock of human capital which is the greatest resource of every organization.

Secondly, the reviews have proven that the impact of COVID-19 on business enterprise has been unique, unprecedented, and may be endless. With new strains and new waves emerging unabated, COVID-19 is peerless when it comes to global health crises that have posed the biggest challenge to the CSR of firms. The momentum of infections across the globe and the seasonal wave with various mutated strains makes it difficult to predict the future of COVID-19 and range of business disruptions with clarity and certainty. This notwithstanding business organizations can seize the opportunities created by COVID-19 to develop better risk management strategies, bolster their brand image to obtain social legitimacy, and redesign their supply chains to enhance efficiency.

Our study contributes in two ways to advancing the theories of corporate social responsibility. Firstly, our study contributes to the emerging field of “evolutionary theory of corporate social responsibility” that emerged from Darwin’s analogy that the most adaptive species are the fittest. In this case, we contend that only adaptive enterprises that are fit can survive COVID-19 and other pandemics irrespective of how much funds they invest in CSR. These are the enterprises who earliest in time see the risks posed by COVID-19 and similar pandemics on business operations. These enterprises have the systems to see things clearly and weigh them justly. They then apply their experience to succeed, not merely because they have an innate power but because the impact of COVID-19 is so rapid and the accompanying competition so fierce that the enterprise that makes a late start is left out and can seldom overtake others. Evolutionary theory of CSR teaches enterprises to develop CSR strategies that go beyond simply designing plans to mitigate damages when crises such as COVID-19 occur. Instead, CSR must include the development of a robust and continuous information sensing system that constantly feed enterprises with complete, timely correct, relevant updates on potential changes in the environment that threaten business stability.

The study also contributes to advancing the frontiers of behavior theory of CSR. Behavioral CSR theorists have stoked a new controversy in their analysis of the impact of COVID-19 on CSR. They argue that COVID-19 CSR-related reactions and interventions are only transient and will not necessarily lead to positive organizational outcomes. They contend that CSR positive outcomes will occur only if CSR is continuously embedded within the organizational structure and strategy. Thus, an enterprise that seeks to boost their economic fortunes through extended CSR during COVID-19 may find their actions mired in chaos and confusion.

Typical of academic studies, a number of limitations may affect the results of this research. For example, the studies were taken from only ten databases and supplemented with three additional sources. This implies that all other studies outside these sources were ignored. The small sample size of articles studied may limit the findings of this research. Relatedly, the strict inclusive and exclusive criteria used to select articles means that other articles with potentially useful information were deemed lower-quality, downgraded, and disregarded. Further, the methodological limitations of the parent studies (particularly, regarding the sampling strategies of reviewed materials in the case of primary studies) limits the findings of the research. This is because most of these studies did not clearly indicate how participants in the studies were recruited and sampled and that may limit the transferability of the findings of this research. Even in the case of the secondary research-based studies, the authors themselves have disclosed limitations regarding the process of sampling the studies which further limits any analysis made from them.

This study included only articles published in the English language and the coverage of the final set of admitted articles did not equally cover all the geographical areas of the world. This limits the generalizability of the findings to other contexts. This review is primarily a tangential contribution to the overall fight against COVID-19 from an organization point of view. Future research must relook at the proposed compulsory unemployment insurance and their ability in ameliorating the effect of future pandemics. This study requires more primary-based information that can be simulated to understand the different scenarios of effectiveness based on historical records and projections into the future.

## Figures and Tables

**Table 1:** Summary of CSR-related global health crisis extracted from databases and other sources.

Databases	HIV-AIDS Pandemic	Environment Health Pandemic	Spanish Flu	Cholera	Opioid Epidemic	Malaria	Obesity Epidemic	COVID-19 Pandemic
Web of Science	204	342	17	35	32	109	21	25
SCOPUS	193	161	9	17	13	28	9	17
EBSCO	68	47	7	12	19	39	11	23
Pro-Quest	17	21	2	8	5	31	16	18
Directory of Open Access Journals	86	81	8	19	39	52	7	25
Digital Library of the Commons Repository	73	75	5	41	28		31	23
Education Resources Information Center	101	108	18	23	20	38	4	32
Social Science Research Network	93	106	6	19	6	64	6	23
Public Library of Science	98	121	12	36	3	43	9	21
arXiv e-Print Archive	121	106	6	3	9		6	19
Social Science Research Network	108	79	9	8	78	52	18	31
Google Scholar	74	54	4	13	23	19	15	29
Others	17	15	5	9	8	48	9	12

**Table 2:** Descriptive characteristics of extracted articles for systematic analysis.

Study	Year of Publication	Methodological Design	Setting	Focus	Funding	Key Objectives
Gentilini	2020	Quantitative	Global	COVID/MES/Cholera	Yes	How countries and Multinational Companies (MNCs) are responding to the COVID-19 pandemic, Cholera/MES
Lindgreen et al.	2009	Quantitative	Botswana and Malawi	Global Health/COVID/MES	Yes	Economic benefits of health-related CSR practices
Amoako et al.	2019	Quantitative	Ghana	Environment and Health/Spanish Flu/opioid	Yes	Health-related CSR activities among the oil marketing companies
Makwara et al.	2019	Quantitative	Zimbabwe and South Africa	HIV-AIDS/Spanish Flu/Malaria/Cholera	Yes	Employee’s HIV and AIDS-related corporate social responsibility (CSR) practices by small business based on experiences from the Spanish Flu/Malaria
Flanagan and Whiteman	2007	Quantitative	Brazil	HIV-AIDS/COVID/Spanish Flu/Malaria	Yes	Private and public Partnership to fight HIV-AIDS based on experiences from the Spanish Flu/Malaria
Utuk et al.	2017	Quantitative	Nigeria	HIV-AIDS/COVID/Spanish Flu/Malaria	Yes	Stigmatising attitudes towards co-workers with HIV in the workplace based on experiences from the Spanish Flu/Malaria
uduji et al.	2019	Quantitative	Nigeria	HIV-AIDS	Yes	Impact of CSR of multinational oil companies on HIV/AIDS prevalence in Nigeria based on experiences from Cholera/Malaria
Bowen et al.	2014	Qualitative	South Africa	HIV-AIDS/COVID/Spanish Flu/Malaria	Yes	Guidelines for effective workplace HIV/AIDS intervention management by construction firms based on experiences from Cholera/Malaria
Rampersad	2013	Qualitative	South Africa	HIV-AIDS/COVID/	Yes	Moral and social responsibility of the corporate sector in its effort to deal with the issue of HIV/AIDS
Ferreira	2002	Qualitative	Global	HIV-AIDS	Yes	Access to affordable HIV/AIDS drugs: The human rights obligations of multinational pharmaceutical corporations based on experiences from Cholera/Malaria
Bolton	2002	Qualitative	South Africa	HIV-AIDS	Yes	How South African companies are taking action against HIV in ways that set new benchmarks
Mahajan et al.	2007	Qualitative	Southern Africa	HIV-AIDS	Yes	An overview of HIV/AIDS workplace policies and programmes in southern Africa based on experiences from Cholera/Malaria
Davis and Anderson	2008	Qualitative	Global	HIV-AIDS/COVID/Spanish Flu/Malaria	Yes	Demands faced by multinationals to assume greater responsibility for solving social problems large and small.
based on experiences from Cholera/Malaria Dufee	2006	Qualitative	Global	HIV-AIDS	Yes	Corporate Responsibility and the AIDS Catastrophe in Sub-Saharan Africa, Pharmaceutical companies
Stadler	2004	Qualitative	South Africa	HIV-AIDS	Yes	Health-related corporate social responsibility initiatives in commercial advertising agency
Rajak	2010	Qualitative	South Africa	HIV-AIDS	Yes	How relations between employer and employee are being transformed by corporate HIV programmes
Sharma and Kiran	2012	Qualitative	India	HIV-AIDS/COVID/Spanish Flu/Malaria	Yes	The status and progress and initiatives made by large firms of India in context to CSR policy framing and implementation
Orlitzky et al.	2011	Qualitative	Global	Environment and Health/opioid/obesity	Yes	Agenda for future research on strategic CSR and environmental sustainability based on experiences from Cholera/Malaria
Lyon and Maxwell	2008	Qualitative	Global	Environment and Health opioid/obesity	Yes	The motives for and welfare effects of environmental corporate social responsibility (CSR) based on experiences from Cholera/Malaria/Obesity/opioid crises
Sanyal and Neves	2001	Qualitative	Global	Environment and Health opioid/obesity	Yes	The Valdez Principles
Welker	2009	Qualitative	Indonesia	Environment and Health opioid/obesity	Yes	The corporate social responsibility industry, and environmental advocacy in Indonesia
Shaukat et al.	2016	Qualitative	Global	Environment and Health opioid/obesity	Yes	Board Attributes, Corporate Social Responsibility Strategy, and Corporate Environmental and Social Performance
Coussens and Harrison	2007	Qualitative	Global	Environment and Health/Spanish Flu/Cholera/Malaria	Yes	Global Environmental Health in the 21st Century
Málovics et al.	2008	Qualitative	Global	Environment and Health	Yes	The role of corporate social responsibility in strong sustainability
Kulczycka et al.	2016	Qualitative	Global	Environment and Health/Spanish Flu/Cholera/Malaria	Yes	Communication about social and environmental disclosure by large and small copper mining companies
Reinhardt and Stavins	2010	Qualitative	Global	Environment and Health	Yes	Corporate social responsibility, business strategy, and the environment
Chandler	2020	Qualitative	Global	Environment and Health/Spanish Flu/Cholera/Malaria	Yes	Reflecting on the need to include CSR principles in future legislative reforms
Kolk	2016	Qualitative	Global	Environment and Health	Yes	The environmental responsibility of international business
Alvarado-Herrera	2017	Qualitative	Global	Environment and Health/Spanish Flu/Cholera/Malaria	Yes	A scale for measuring consumer perceptions of corporate social responsibility following the sustainable development paradigm
Schönherr et al.	2018	Qualitative	Global	Environment and Health	Yes	How the Sustainable Development Goals (SDGs) as a global agenda may serve as a reference framework to support TNCs in improving their corporate social responsibility (CSR) engagement
Xia et al.	2018	Qualitative	Global	Environment and Health	Yes	Conceptualising the state of the art of corporate social responsibility (CSR) in the construction industry and its nexus to sustainable development
Givel	2017	Qualitative	Global	Environment and Health/Spanish Flu/Cholera/Malaria	Yes	The primary goal of the Responsible Care effort to change public concerns and opinion about chemical industry environmental and public health practices
Alon et al.	2020	Qualitative	Global	COVID	Yes	The Impact of COVID-19 on Gender Equality
Francis and Pegg	2020	Qualitative	Nigeria	COVID/Spanish Flu/Cholera	Yes	The challenges that one long running micro-scale development project has faced due to the COVID 19 disease outbreak and the closure of all schools in Rivers State, Nigeria
Williamson et al.	2020	Qualitative	Global	COVID	Yes	COVID-19 and experiences of moral injury in front-line key workers
Vaccaro et al.	2020	Qualitative	US	COVID	Yes	Practice Management During the COVID-19 Pandemic
Shingal	2020	Qualitative	Global	COVID/Spanish Flu/Cholera	Yes	Services trade and COVID-19
Boone et al.	2020	Qualitative	Global	COVID	Yes	The socio-economic implications of the coronavirus and COVID-19 pandemic
Hevia and Neumeyer	2020	Qualitative	Global	COVID	Yes	A Conceptual Framework for Analyzing the Economic Impact of COVID-19 and its Policy Implication
Zeren and Hizarci	2020	Qualitative	Global	COVID	Yes	The Impact of COVID-19 Coronavirus on Stock Markets based on experiences from MES/Malaria
Cabral and Xu	2020	Qualitative	Global	COVID	Yes	Seller Reputation and Price Gouging: Evidence from the COVID-19 Pandemic
Delwin et al.	2019	Qualitatve	SubSaharan Africa	HIV-AIDS	Yes	Role of Multinationals in HIV-AIDS in Asian/SubSahara based on experiences from Cholera/Malaria
Dickson and Stevens	2005	Quantitative	South Africa	HIV-AIDS	Yes	Understanding the response of large South African companies to HIV/AIDS
Bendel	2003	Quantitative	Global South	HIV-AIDS	Yes	Response of large corporations to HIV/AIDS in Southern Africa based on experiences from Cholera/Malaria
Ntim	2016	Quantitative	subsaharan	HIV-AIDS	Yes	HIV/AIDS disclosures in Sub-Saharan Africa based on experiences from Cholera/Malaria
Delmas et al.	2013	Quantitative	Global	Environment and Health	Yes	Socially responsible investing
Annan-Diab	2017	Quantitative	US	Environment and Health	Yes	The importance of adopting an interdisciplinary approach to education for sustainable development based on experiences from Cholera/Malaria
Suárez-Cebador	2018	Quantitative	Portugal	Environment and Health	Yes	A model to measure sustainable development in the hotel industry
Chuang and Huang	2018	Quantitative	Taiwan	Environment and Health	Yes	The Effect of Environmental Corporate Social Responsibility on Environmental Performance and Business Competitiveness
Marco-Fondevila	2018	Quantitative	Spain	Environment and Health/COVID/Spanish Flu/Cholera	Yes	The determinants and empirical interrelations between accountability standards and environmental proactivity
López-Pérez	2017	Quantitative	South America	Environment and Health	Yes	Analysis of specific corporate social responsibility +CSR) training in sustainable development to boost the potential impact of CSR on shareholder value
Taylor et al.	2018	Quantitative	Global	Environment and Health	Yes	Benefits associated with voluntary disclosure of corporate social responsibility (CSR) activities
Osmani	2019	Quantitative	China	Environment and Health COVID/Spanish Flu/Cholera	Yes	Corporate Social Responsibility for Sustainable Development in China. Recent Evolution of CSR Concepts and Practice within Chinese Firms based on experiences from Cholera/Malaria
Dimmler	2017	Quantitative	South Africa	Environment and Health COVID/Spanish Flu/Cholera	Yes	Linking social determinants of health to corporate social responsibility: Extant criteria for the mining industry based on experiences from Cholera/Malaria
Senay and Landrigan	2018	Quantitative	US	Environment and Health COVID/Spanish Flu/Cholera	Yes	Assessment of environmental sustainability and corporate social responsibility reporting by large health care organizations
Albuquerque et al.	2020	Quantitative	US	COVID/Environment	Yes	How firms with high Environmental and Social (ES) ratings fare during the first quarter of 2020 compared to other firms
Shan and Tang	2020	Quantitative	China	COVID	Yes	The role of employee satisfaction in withstanding the public health shock
Laing	2020	Quantitative	Global	COVID	Yes	The economic impact of the Coronavirus 2019 (COVID-2019): Implications for the mining industry
Makridis and Hartley	2020	Quantitative	US	COVID	Yes	The Cost of COVID-19: A Rough Estimate of the 2020 US GDP Impact
Nuno-Fernandes	2020	Quantitative	Europe	COVID	Yes	Economic effects of coronavirus outbreak (COVID-19) on the world economy
Maital and Barzani	2020	Quantitative	Global	COVID	Yes	Global Economic Effects of COVID-19
Barua	2020	Quantitative	Global	COVID	Yes	The Economic Implications of the Coronavirus (COVID-19) Pandemic
Johson et al.	2010	Qualitative	Global	Natural Disasters	Yes	Reasons why MNC engage in health-related CSR
Vian et al.	2007	Qualitative	Global	Global Health	Yes	How multinational pharmaceutical companies engage in CSR activities in the developing world
Soobaroyen and Ntim	2013	Qualitative	South Africa	HIV-AIDS COVID/Spanish Flu/Cholera	Yes	Global Reporting Initiative guidelines on HIV/AIDS to assess on whether corporations have adopted a substantive management strategy
Long	2016	Qualitative	Tanzania	HIV-AIDS COVID/Spanish Flu/Cholera	Yes	Role of PEPFAR Tanzania pin the national health sector’s HIV/AIDS policy shift based on experiences from Cholera/Malaria
Gilbert	2017	Qualitative	South Africa	HIV-AIDS COVID/Spanish Flu/Cholera	Yes	Investigating HIV/AIDS intervention management by construction organizations in South Africa based on experiences from Cholera/Malaria

**Table 3:** MMAT assessment of quality of extracted articles for systematic review.

**Quantitative Research**	**Bowen et al.**	**Rampersad**	**Ferreira,**	**Bolton**	**Mahajan et al.**	**Davis and Anderson**	**Dufee**	**Stadler**	**Rajak**	**Sharma and Kiran**	**Orlitzky et al.**	**Lyon and Maxwell**	**Sanyal and Neves**	**Welker**	**Shaukat et al.**	**Coussens and Harrison**	**Málovics et al.**	**Kulczycka et al.**	**Reinhardt and Stavins**	**Chandler**	**Kolk**	**Alvarado-Herrera**	**Schönherr et al.**	**Xia et al.**	**Givel**	**Alon et al.**	**Francis and Pegg**	**Williamson et al.**	**Vaccaro et al.**	**Shingal**	**Boone et al.**	**Hevia and Neumeyer**	**Zeren and Hizarci**	**Cabral and Xu**	**Delwin et al.**
Data sources relevant?	Y	Y	Y	Y	Y	Y	Y	Y	Y	Y	Y	Y	Y	Y	Y	Y	Y	Y	Y	Y	Y	Y	Y	Y	Y	Y	Y	Y	Y	Y	Y	Y	Y	Y	Y
Data analysis process relevant?	Y	Y	Y	Y	Y	Y	Y	Y	Y	Y	Y	Y	Y	Y	Y	N	Y	Y	Y	Y	Y	Y	Y	N	N	Y	Y	Y	Y	Y	Y	N	Y	Y	Y
Findings relate to context?	Y	Y	Y	N	Y	Y	Y	Y	Y	Y	Y	Y	Y	Y	Y	Y	Y	Y	N	Y	Y	Y	Y	Y	N	Y	Y	Y	Y	Y	Y	N	Y	Y	Y
Findings relate to researchers’ influence?	N	N	N	N	Y	N	N	N	N	N	N	N	N	N	N	N	N	N	N	N	N	N	N	N	N	N	N	N	Y	N	N	N	N	N	Y
Clear description of the sampling process of respondents	Y	Y	Y	Y	Y	Y	Y	Y	Y	Y	Y	Y	Y	Y	Y	N	Y	Y	Y	Y	Y	Y	Y	Y	Y	Y	Y	Y	Y	Y	N	Y	Y	Y	Y
Support from funding agencies	N	N	N	N	N	N	N	N	N	N	N	N	N	N	N	N	N	N	N	N	N	N	N	N	N	N	N	N	N	N	N	N	N	N	N
	67%	67%	67%	50%	83%	67%	67%	67%	50%	67%	67%	67%	67%	67%	67%	33%	67%	67%	50%	67%	67%	67%	67%	67%	33%	67%	67%	67%	83%	67%	50%	33%	67%	67%	83%
**Qualitative Research**	**Dickson and Stevens**	**Bendel**	**Ntim**	**Delmas et al.**	**Annan-Diab**	**Suárez-Cebador**	**Chuang and Huang**	**Marco-Fondevila**	**López-Pérez**	**Taylor et al.**	**Osmani**	**Dimmler**	**Senay and Landrigan**	**Albuquerque et al.**	**Shan and Tang**	**Laing**	**Makridis and Hartley**	**Nuno-Fernandes**	**Maital and Barzani**	**Barua**	**Johson et al.**	**Vian et al.**	**Soobaroyen and Ntim**	**Long**	**Gilbert**	**Gentilini**	**Lindgreen et al.**	**Amoako et al.**	**Makwara et al.**	**Flanagan and Whiteman**	**Utuk et al.**	**Uduji et al.**			
Clear description of the randomization?	Y	Y	Y	Y	Y	Y	Y	Y	Y	Y	Y	Y	Y	Y	N	Y	Y	Y	Y	Y	Y	N	Y	Y	Y	Y	Y	Y	Y	Y	Y	Y			
Clear description of allocation or concealment?	Y	Y	Y	Y	Y	Y	Y	Y	Y	Y	Y	Y	Y	Y	Y	Y	Y	Y	N	Y	Y	N	Y	Y	Y	Y	Y	Y	Y	Y	N	Y			
Complete outcome data?	Y	Y	Y	Y	Y	Y	Y	N	Y	Y	Y	Y	Y	Y	N	Y	Y	N	Y	Y	N	Y	Y	Y	Y	N	Y	Y	Y	Y	Y	Y			
Low withdrawal/drop-out?	Y	Y	Y	Y	Y	Y	Y	Y	Y	Y	Y	Y	Y	Y	Y	Y	Y	Y	Y	Y	N	Y	Y	Y	Y	N	Y	Y	Y	Y	Y	Y			
Reason for studying specific organizations	Y	N	Y	N	N	N	N	N	Y	N	N	Y	N	Y	N	Y	N	N	Y	Y	N	Y	Y	N	Y	Y	N	Y	N	N	Y	N			
The influence of the organization on the research	N	N	N	N	N	N	N	N	N	N	N	N	N	N	N	N	N	N	N	N	N	N	N	N	N	N	N	N	N	N	N	N			
Researcher influence in qualitative and mixed methods studies	N	N	N	N	N	N	N	N	N	N	N	N	N	N	N	N	N	N	N	N	N	N	N	N	N	N	N	N	N	N	N	N			
Support from funding agencies	Y	Y	Y	N	Y	Y	N	Y	Y	N	Y	Y	N	Y	N	N	Y	Y	Y	N	N	Y	Y	Y	Y	N	Y	Y	Y	Y	Y	Y			
Total score (%)	75%	63%	75%	75%	75%	75%	75%	50%	75%	75%	75%	75%	50%	75%	25%	75%	75%	50%	63%	75%	25%	50%	75%	75%	75%	38%	75%	75%	75%	75%	63%	75%			

Key: Y = Yes, N = No.

**Table 4:** Differences and similarities between the global health pandemics that have influenced CSR.

Variables	COVID-19	Spanish Flu	HIV-AIDS	Cholera	Environmental Pollution	Malaria	MES	Ebola	Opioid	Obesity
Scope of geographical coverage of infection	All Continents/Countries	Asia/Europe/America	Mostly Sub-Saharan Africa	Underdeveloped countries	All Continents/Countries	Underdeveloped countries	Middle East	Africa	Developed Countries	Developed Countries
Scale of public panic reaction to disease	Very High	Very High	Very High	High	Low	High	High	High	Low	Low
Seasonal variation of infection	Yes	Yes	No	High	Low	High	High	High	Low	Low
Need for physical lockdowns to control infections	Yes	Yes	No	No	No	No	No	No	No	No
Isolation of patients to control infection	Yes	Yes	Yes	Yes	No	Yes	Yes	Yes	No	No
Scale of stigma associated with infection	High	High	Very High	None	None	None	High	Very High	High	High
Scale of conspiracy theories to explain infection	Yes	Yes	Yes	No	No	No	Yes	Yes	No	No
Effect of mass gathering on infections	Yes	Yes	No	Yes	No	Yes	Yes	Yes	No	No
Possibility of early detection and treatment	Yes	Yes	Yes	Yes	Yes	Yes	Yes	Yes	Yes	Yes
Effect of underlining conditions of criticality of illness	Yes	Yes	No	Yes	No	Yes	Yes	Yes	Yes	No
Age variation in infection rate	Elderly	Elderly	Youth	All	All	All	All	All	All	All
Gender variation in infection rate	Non	Non	Non	Non	Non	Non	Non	Non	Non	Non
Geographical concentration of highest rate of infection/deaths	Advanced/Emerging Countries	Advanced/Emerging Countries	Developing Countries	Developing Countries	All Continents/Countries	Developing Countries	Middle East	Africa	Advanced/Emerging Countries	Advanced/Emerging Countries
Scale of frontline deaths	Very High	Very High	Low	High	Low	High	High	High	Low	Low
Scale of direct impact of epidemic on socio-economic activities globally	Very High	Very High	High	Low	High	Low	High	High	Medium	Medium
Scale of direct impact of epidemic on cost to businesses globally	Very High	Very High	Very High	Low	High	Low	High	High	High	High
Scale of direct impact of epidemic on business revenue globally	Very High	Very High	Medium	Low	Medium	Low	Medium	Medium	Low	Low
Scale of use of inter-government regulations to control infection	Very High	Very High	High	Medium	High	Medium	Medium	High	Low	Low
Epidemic disruptions as cause of major employee layoffs	Very High	Very High	Medium	Low	Low	Low	Low	Low	Low	Low
Cross border lockdowns to prevent spread of epidemics	Yes	Yes	No	No	No	No	Yes	Yes	No	No
Rate of infection among people	Very High	Very High	Low	High	Low	High	Very High	Very High	Low	Low
Mode of transmission of epidemic	Droplets	Droplets	Blood	Sanitation	Prolong Exposure	Sanitation	Droplets	Droplets	Habits	Habits
Intensity of CSR	Very High	Very High	Very High	Very High	Very High	Very High	Low	Low	Low	Low
Criticisms of CSR	Very High	Very High	Very High	Very High	Very High	Very High	Very High	Very High	Very High	Very High

## Data Availability

The data for this research is held by the authors and will be made available upon reasonable request.
